# Screening for metabolic syndrome risk factors in mestizo, tarahumara and mennonite scholars from Chihuahua Mexico

**DOI:** 10.1186/1753-6561-6-S3-P31

**Published:** 2012-06-01

**Authors:** I Leal-Berumen, V Santana-Rodriguez, P Hernández-Rodríguez, V Moreno-Brito, A Licón-Trillo, E González-Rodríguez, I Alcalá-Sánchez, M Conchas-Ramírez, C Santiago-Antonio

**Affiliations:** 1 Multidisciplinary Metabolic Syndrome Study Group, Universidad Autónoma de Chihuahua, Chihuahua, 3100, Mexico; 2 Colegio de Nutriólogos, Chihuahua, 3112, Mexico

## Background

Obesity and diabetes mellitus prevalence has increased during the past decade among adults and adolescents in Mexico. According to la Encuesta Nacional de Salud y Nutrición 2006, 30% of adult population is obese and 39% has overweight, whereas in adolescents is close to 31%. Diabetes prevalence is estimated to be 14.4% in adult Mexican population [1]. Metabolic syndrome (MS) in adults is defined as a cluster of risk factors including abdominal obesity, dyslipidemia, glucose intolerance and hypertension, the presence of three or more components increases the risk for heart disease, diabetes mellitus type 2 and obesity [2,3]. Early identification of children at risk of developing MS must be estimated.

## Objective

To screen for MS risk factors among mestizo, tarahumara and mennonite teenagers from Chihuahua, Mexico.

## Materials and methods

A convenient study was performed in high schools from Chihuahua small towns (Guachochi, Cuauhtemoc and Carichí) including 544 teenage students from 12-19 years old, 42% males, 58% females. Blood pressure, anthropometric measures, fasting glucose, triglycerides and cholesterol HDL were obtained with signed parental informed consent. We used an adapted MS definition [4].

## Results

In total population 2.6% had abnormal abdominal obesity, 2.2% had increased fasting glucose, 24.6% had abnormal triglycerides, and 29.4% low HDL cholesterol levels. Within populations, triglyceride abnormal levels where observed in 35% tarahumaras, in 26% mestizos and in 12.9% mennonites, being greater in females. HDL cholesterol abnormal levels where observed in 55.4% tarahumaras, in 23.3% mestizos and in 12.3% mennonites. Abnormal blood pressure was mainly detected in mennonites. According to BMI female percentile classification, tarahumaras showed greater overweight (20.2%), mestizas greater obesity (11.4%) and 15.4% male tarahumaras were overweight.

## Conclusions

This is the first study in Chihuahua that looks for MS risk factors among scholars from different ethnicity. In general, tarahumaras seem to be at higher risk to develop MS, females from this population, had greater overweight, as well as abnormal triglycerides and HDL cholesterol levels. Mennonites were healthier than mestizo and tarahumara teenagers. Our results imply that cultural habits and genetic play an important role in developing MS risk factors that we need to consider when prevention strategies are designed.
